# Right aortic arch with an aberrant left subclavian artery and aortic coarctation including a descending aortic aneurysm

**DOI:** 10.1186/s13019-019-0878-y

**Published:** 2019-04-02

**Authors:** Frantisek Sabol, Peter Candik, Adrian Kolesar, Tomas Toporcer

**Affiliations:** 1 Clinic of Cadiac Surgery, Medical Faculty of P.J.Safarik University and Eastern Slovak Institute for Cardiovascular Diseases Ltd, Kosice, Slovakia; 2 Clinic of Anaesthesiology and Intensive Medicine, Medical Faculty of P.J.Safarik University and Eastern Slovak Institute for Cardiovascular Diseases Ltd, Ondavska 8, 040 01 Kosice, Slovakia

**Keywords:** Aortic coarctation, Partial cardiopulmonary bypass, Right aortic arch

## Abstract

**Backround:**

The right aortic arch and aortic coarctation are rare congenital anomalies with the incidence of 0.1% and 0.03–0.04%. We present a case report of a 51-year-old woman with the right aortic arch with aberrant left subclavian artery and coarctation of the aorta with post-stenotic aneurysm.

**Case presentation:**

Resection of the coarctation and aneurysm with replacement by tubular prosthesis was performed on partial cardiopulmonary bypass via femoral vessels.

**Conclusion:**

Partial cardiopulmonary bypass is an applicable method for ensuring the perfusion of the distal part of the body and an aberrant left subclavian artery is not a contraindication of this technique.

## Introduction

Asymptomatic congenital anomalies with an incidence of 0.5–3% include left aortic arch with an aberrant right subclavian artery with or without diverticulum of Kommerell, double site ligamentum arteriosum or ductus arteriosus and left circumflex aorta [1, 2]. Right aortic arch (RAA) usually comprises an aberrant left subclavian artery most commonly with retroesophageal diverticulum. It is often associated with left sided ductus arteriosus and vascular ring [1]. Symptomatic aortic arch anomalies contain hypoplasia, coarctation (CoA) and pseudocoarctation of the aorta [1]. Aortic arch anomalies can occur in an isolation and with other congenital anomalies as well.

In 1948, Edwards explained the various congenital anomalies by system of embryonic double aortic arch and site of interruption or atresia of the embryonic arch system [2]. RAA occurs in 0.1% of adults [2]. Regression of 4th left aortic arch can occurs in different part of the arch with different finally anatomy composition (Fig. 2a and b). On the other hand, CoA presents 5–8% of all congenital heart defects with estimated incidence of 0.3–0.4 per 1000. It can by solitary in 82% of humans or associated with other congenital malformations and symptoms. Late aneurysmal formation in the distal CoA is one of the complications due to untreated CoA with high risk of aortic rupture and death [3].

## Case report

**Fig. 1:**
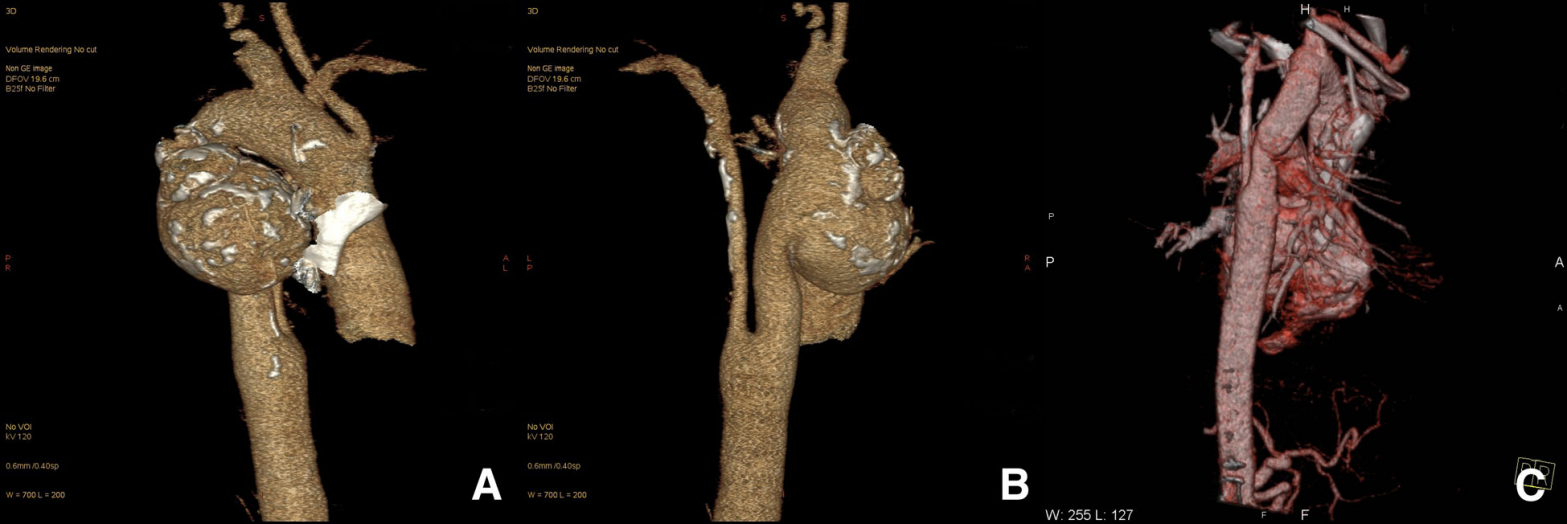
CT depiction of RAA and aneurysmal formation and the descending aorta after surgery (**a** – dorsal view before surgery, **b** – descending aorta after surgery)

A 51-year-old woman with history of RAA in surveillance attended a cardiologist due to dyspnea and palpitation. The patient underwent a computer tomography (CT) evaluation which confirmed RAA with a common origin of both carotid arteries, a separate origin of the right subclavian artery, coarctation of the aorta with the diameter of 12 mm, an aortic aneurysm below the coarctation with the diameter of 60 mm and origin of the left subclavian artery right below the aortic aneurysm at the level of the 6th thoracic vertebra (Fig. 1a). The patient was admitted to our clinic and echocardiography revealed the left ventricle ejection fraction of 60%, without any other cardiac malformations. The diameter of the left common carotid artery was 5 mm. Coronary vessels angiography did not show any stenosis or other malformations. Because of the diameter of the aneurysm (as a new CT finding), surgery was recommended.

**Fig. 2:**
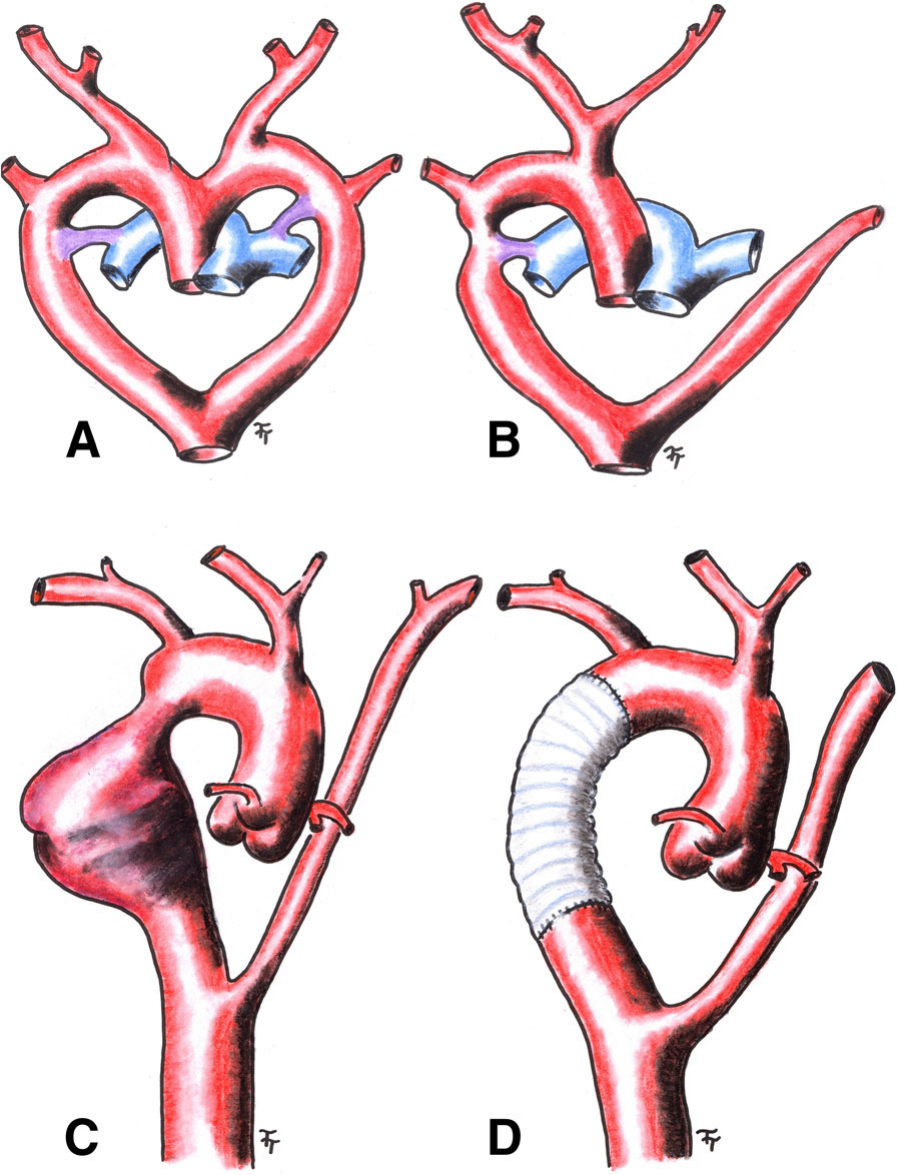
Sketch of an embryonic development of the RAA and an aorta before and after surgery in presented case (**a** – before and **b** after a regression of the left aortic arch between the left common carotid artery and the left subclavian artery, blue – pulmonary trunk and pulmonary arteries, purple – arterial duct, red – aorta and arteries; **c** – presented case before surgery; **d** – presented patient after surgery)

**Fig. 3:**
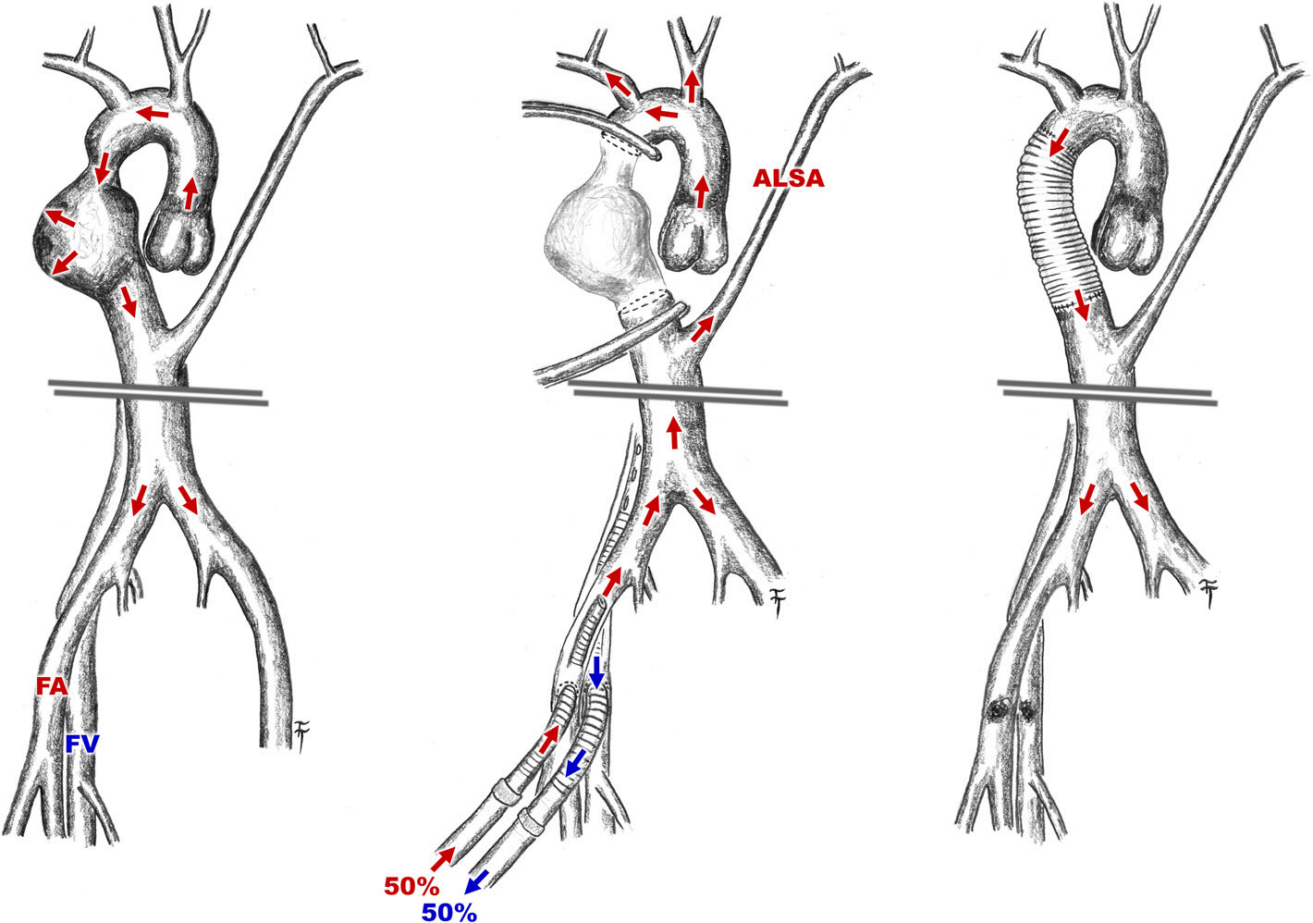
Sketch of the resection of an aortic coarctation and aneurysm, partial extracorporeal circulation via femoral vessels for lower part of body and left upper limb perfusion was applicated, resected part of the right descending aorta was replaced by tubular prothesis (ALSA – aberrant left subclavian artery; FA – femoral artery; FV – femoral vein)

Partial cardiopulmonary bypass (CPB) via femoral vessels was established. Right posterolateral thoracotomy in the 4th intercostal space was done (Fig. 3). Ventilation of the left lung was maintained by selective intubation. The thoracic aorta was X-clamped just above the aneurysm while another (distal) clamp was placed right below the aneurysm and also above the aberrant left subclavian artery. Thus, perfusion and oxygenation of the aortic arch branches were provided by the beating heart as well as by selective lung ventilation. Perfusion of the thoracic and abdominal aorta below the distal clamp was secured by a partial CPB (2 l/min) while the adequate perfusion was controlled and adjusted by comparison of arterial pressure on the both radial and left femoral arteries. The aortic coarctation and aortic aneurysm were resected and replaced with tubular prosthesis of 22 mm in diameter (Figs. 2d and 3). The CPB time lasted 94 min in a mild hypothermia (34 degrees Celsius).

The postoperative course was uneventful. On the 8th postoperative day the patient was discharged (Fig. 1b).

## Discussion

In a normal situation of the left aortic arch, the right 4th primitive aortic arch forms a brachiocephalic trunk while the left 4th primitive arch converts into the definitive aortic arch. The left common carotid artery and the left subclavian artery are formed from the 3rd left primitive aortic arch and 7th left intersegmental artery. In an situation of RAA, right 4th primitive aortic arch forms the definitive RAA, the 5th primitive arches and right 4th primitive arch regress, the 3rd left arch forms the left common carotid artery and the left dorsal primitive aorta forms the diverticulum of Kommerell with an aberrant retroesophageal left subclavian artery as a result of 7th left intersegmental artery. This pathology is known as RAA with aberrant left subclavian artery arising from the retroesophageal diverticulum [1]. In the presented case the retroesophageal diverticulum of Kommerell was not formed, which is very rare (Fig. 2a and b). Furthermore, CoA of RAA together confirm a persistence of the right-sided ductus arteriosus developed during the prenatal period from the distal part of the 6th right primitive aortic arch. The disappearance of the ductus arteriosus on the right side has ensured no formation of a vascular ring and asymptomatology of the anomalies.

In general, three types of surgery are available. They consist of: a) resection of the diseased aortic part and simple end-to-end suture; b) resection of CoA and its replacement with a tubular graft and c) extra-anatomical bypass [4]. Resection with the end-to-end anastomosis is more suitable in infant patients because of absence of a no-growing graft and higher mobility and elasticity of tissues as well [4]. Extra-anatomical bypass has more benefits for patients with CoA recurrence without aneurysm formation because of adhesions after a previous surgery [4]. Resection of CoA with graft replacement is indicated for more extensive and severe stenotic CoA and CoA combined with aneurysmal formation. The disadvantage of this procedure is a prolonged cross-clamp time due to two anastomoses. In addition, a method of the spinal cord and visceral organs protection might be needed as well.

Some authors use full CPB with hypothermic circulatory arrest, Gott’s passive shunt or intraluminal shunt [4, 5]. Partial CPB appears as a convenient and practical method for this type of surgical treatment. Moreover, application of partial CPB goes along with a low risk of spinal and lower body ischemia and provides enough time for anastomoses [6]. Nakamura et al. presented three CoA cases with usage of partial CPB during the surgery. CPB was established via femoral vessels. All three patients underwent a CoA resection with tubular replacement through posterolateral thoracotomy. One patient underwent a concomitant aortic valve replacement via median sternotomy and total CPB [4].

## Conclusions

We can conclude that right-sided aortic arch with an aberrant left subclavian artery without left-sided ductus arteriosus do not cause vascular ring and might be absolutely asymptomatic congenital disease. The presence of an aortic coarctation in this case report documents that this congenital malformation include right-sided ductus arteriosus. Right posterolateral thoracotomy is adequate surgical approach for right-sided aortic arch surgery and resection of thoracic descending aorta can by performed with a partial cardiopulmonary bypass without the need of circulatory arrest. Partial cardiopulmonary bypass is suitable in the field of an abberant subclavian artery which allows pressure monitoring on radial artery.

## Abbreviations

CoA: Aortic coarctation
CPB: Cardiopulmonary bypass
CT: Computer tomography
RAA: Right Aortic Arch
